# Detection of *Leptospira* species in bat cadavers, Czech and Slovak Republics

**DOI:** 10.1080/22221751.2022.2117095

**Published:** 2022-09-26

**Authors:** Veronika Seidlova, Petra Straková, Romana Kejíková, Monika Nemcova, Tomáš Bartonička, Jiří Salát, Lucie Dufková, Silvie Šikutová, Jan Mendel, Clifton McKee, Jan Zukal, Jiri Pikula, Ivo Rudolf

**Affiliations:** a Institute of Vertebrate Biology, Czech Academy of Sciences v.v.i., Brno, Czech Republic; b Department of Ecology and Diseases of Zoo Animals, Game, Fish and Bees, University of Veterinary Sciences Brno, Brno, Czech Republic; c Veterinary Research Institute, Department of Infectious Diseases and Preventive Medicine, Brno, Czech Republic; d Department of Botany and Zoology, Masaryk University, Brno, Czech Republic; e Department of Epidemiology, Johns Hopkins Bloomberg School of Public Health, Baltimore, MD, USA

**Keywords:** Bats, Central Europe, emerging zoonoses, *Leptospira*, *Myotis*, *Nyctalus*

## Abstract

Kidney samples from 300 bat cadavers from the Czech and Slovak Republics were tested for *Leptospira* DNA using PCR and sequencing of three genes (*lipL32*, *flab*, and 16S ribosomal RNA). Overall detection rate was 4.7% and two bat species (*Myotis myotis* and *Nyctalus noctula*) were PCR-positive for at least one gene. Detected *Leptospira* sequences were similar to *L. interrogans* and *L. borgpetersenii*, and included a potentially novel species related to *L. weilii*.

European bats are carriers or reservoirs of several zoonotic bacterial pathogens, including *Leptospira* spp. [1]. Using different approaches, *Leptospira* spp. have been detected in more than 50 bat species from different geographical regions, but mainly from the tropics and subtropics [2]. In Europe, the possible role of bats in the epidemiology of leptospirosis remains to be clarified, as the occurrence of *Leptospira* spp. in bats is poorly known. A survey of wildlife conducted by Fennestad and Borg-Petersen [3] in Denmark showed that four bat species had leptospires visible by dark-field microscopy in urine and/or kidney tissue suspensions: *Myotis daubentonii*, *Pipistrellus pipistrellus*, *Nyctalus noctula*, and *Eptesicus serotinus*. They also observed regular excretion of leptospires in bat urine visible by microscopy in three *Nyctalus noctula* kept alive in captivity for up to 20 weeks. Bai et al. [4] detected *Leptospira* DNA via PCR in 25 bat kidney samples from two species, *Myotis blythii* and *Miniopterus schreibersii*, in the Republic of Georgia. Recently, *Leptospira* spp. DNA was detected in urine samples of four bat species from the Czech Republic and Poland: *Barbastella barbastellus*, *Myotis bechsteinii*, *Myotis myotis*, and *Myotis nattereri* [5].

To study the occurrence of *Leptospira* in Central European bats in greater detail, 300 carcasses of 13 bat species were collected by passive surveillance (due to strict protection of bats that prohibit invasive sampling) from 2009 to 2019 at different sites in the Czech Republic and the Slovak Republic (see Appendix 1 for details). For the animals investigated in this study, carcasses of deceased bats found in the Czech Republic were kindly provided by bat researchers and animal rehabilitation centres. Bat species were identified by experienced bat biologists based on morphological traits and available keys [6]. Team members were authorized to handle wild bats according to the Czech Certificate of Competency (No. CZ01341; §17, Act No. 246/1992 Coll.) and all sampling complied with Czech Law No. 114/1992 on Nature and Landscape Protection. Bat samples were frozen at −80°C after collection and this material was processed immediately. Bat kidneys were dissected, homogenized, and DNA extraction was performed using the QIAamp DNA Mini Kit (Qiagen, USA) according to the manufacturer's protocols. Conventional PCR protocols targeting the outer membrane lipoprotein (*lipL32*) gene [4], the 16S ribosomal RNA (rRNA) gene [7], and the flagellin B (*flaB*) [7] were used to detect *Leptospira* DNA. The *lipL32* gene is only present in pathogenic and intermediate *Leptospira* species (lineages P1 and P2 in the classification scheme from Vincent et al. [8]), but *flaB* and 16S rRNA are present in all *Leptospira* lineages. Bidirectional sequencing was performed using an ABI PRISM 3100 Genetic Analyzer (Applied Biosystems, USA). The raw DNA sequences were edited and aligned using the Seqman module in Lasergene v6 (DNASTAR, USA) and manually checked. The BLAST algorithm (http://www.ncbi.nlm.nih.gov/blast) was used to confirm that sequences represented *Leptospira* DNA. A database was compiled consisting of *lipL32* and *flaB* sequences from bats, canonical *Leptospira* species, and newly isolated species from environmental samples [8], then phylogenetic trees were inferred using the maximum likelihood method (see Appendix 2 for details).

In total, 13/290 (4.5%) bats from the Czech Republic and 1/10 (10%) bats from the Slovak Republic were positive for *Leptospira* DNA via amplification and sequencing of one or both the *lipL32* and *flaB* genes (4.7% overall). Attempts to amplify the 16S rRNA gene were unsuccessful with the primers and protocol used [7]. As for bat species, *Leptospira* DNA was amplified in 3/69 *Nyctalus noctula* specimens (4.3%) and 11/187 *Myotis myotis* specimens (5.9%). The detection rate did not differ between these two species (chi-square test of proportions, *p* = 0.633) and sexes (chi-square test of proportions, *Nyctalus noctula p* = 0.090 and *Myotis myotis p* = 0.632), respectively. The remaining 11 species tested were all negative (see Appendix 1). The majority of positive *lipL32* sequences (n = 12/14, 11 from *M. myotis* and one from *N. noctula*) were identical to pathogenic *Leptospira interrogans* (GenBank accession number MT4823) previously detected in urine from *Myotis myotis* in the Czech Republic [5]. Two identical sequences (samples N8 and N45 from *N. noctula*, GenBank accession number OM307661) were phylogenetically distinct from other *Leptospira* species detected in bats and instead clustered with *L. weilii*, *L. mayottensis*, and *L. alexanderi* (Figure 1A); the novel sequences from bats shared 93.1%, 92.2%, and 92.7% sequence identity with these three species, respectively. *Leptospira weilii* was first described from the blood of a human patient in Australia [9], *L. mayottensis* was isolated from blood of leptospirosis patients on the island of Mayotte [10], and *L. alexanderi* was isolated from humans in China [11]. The *flaB* gene was successfully amplified from sample N8 only (*Nyctalus noctula* from Czech Republic), and the sequence showed multiple peaks in the electropherogram, suggesting co-infection with multiple *Leptospira* species. Using the Mixed Sequences Reader tool [12], two separate sequences (major and minor) were identified. The N8 major sequence (GenBank accession number ON552553) shared 94.7% sequence identity with *L. borgpetersenii* and the N8 minor sequence (GenBank accession number ON552554) shared 93.4% sequence identity with *L. interrogans* (Figure 1B).

**Figure 1.**
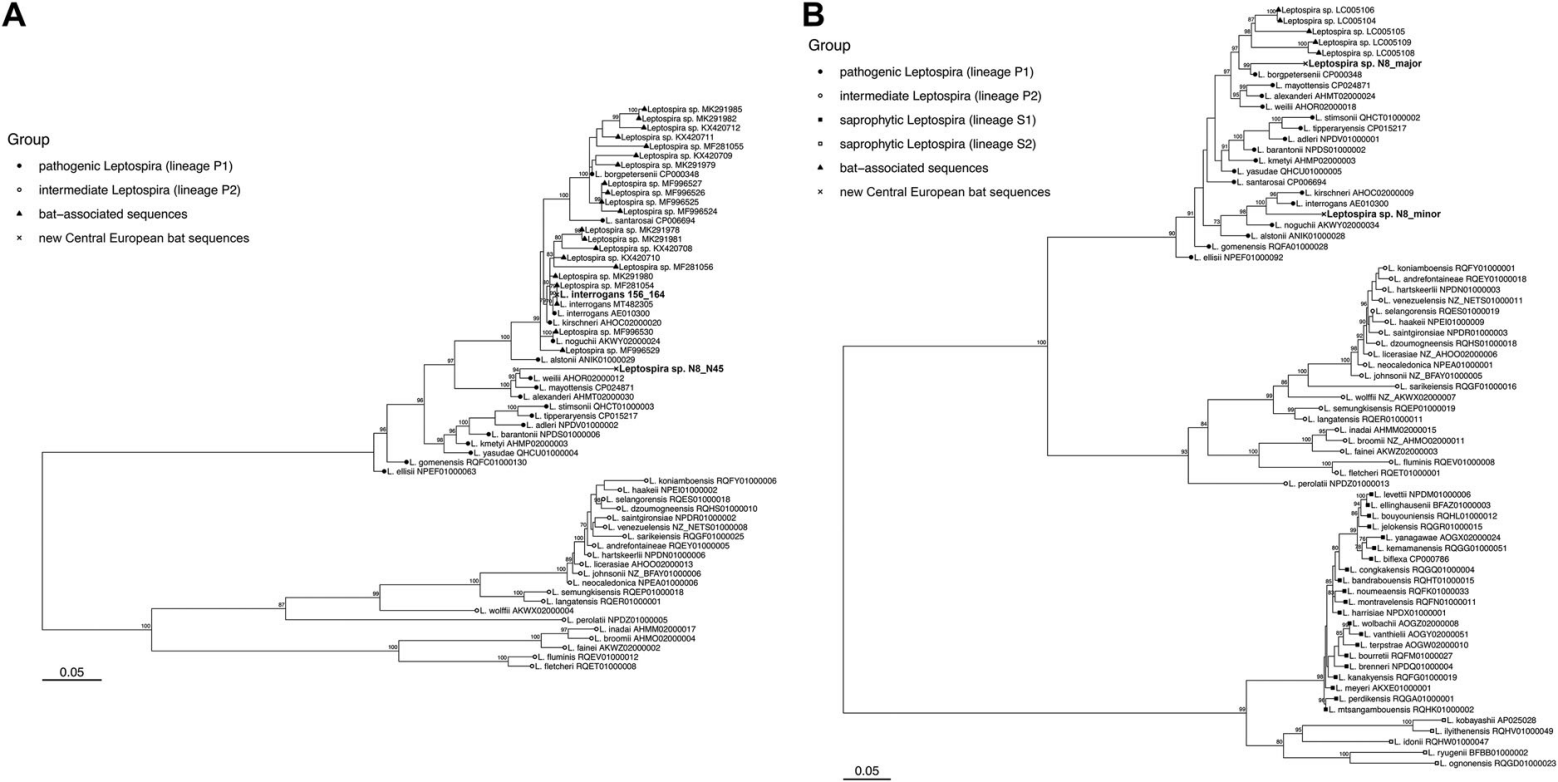
Phylogenetic relationships between *Leptospira lipL32* sequences (A) and *flaB* sequences (B). Separate groups, including new sequences detected in Central European bats, are indicated by distinct symbols. The maximum likelihood trees were inferred using a TIM3+F+I+G4 model for *lipL32* and a TVMe+I+G4 model for *flaB* in IQ-TREE v2.1.1. Numbers next to nodes indicate the percent bootstrap support after 1000 replicates. Branch lengths are in units of substitutions per site.

The presence of *Leptospira* spp. DNA was investigated in kidney tissues of bats from the Czech and Slovak Republics. The overall detection rate (4.7%) is within the range reported from other countries (2–35%; [1]), although the detection rate can be highly variable even in the same species over time, suggesting a possible seasonal pattern of infection in bats that could be influenced by many factors such as sex, roosting behaviour, parturition, lactation, or weaning [13]. While bats have been shown to be hosts of pathogenic *Leptospira* spp. strains, particularly in the tropics and subtropics, the role of European bats in maintaining and shedding *Leptospira* infection, and the potential public health risks from human exposure to bat leptospires remains poorly understood. In this study, we detected three lineages within the pathogenic clade of *Leptospira* (Figures 1A and 1B), one related to *L. borgpetersenii*, one related to *L. interrogans*, and another more distantly related to *L. weilii*, in kidney samples from bat cadavers. While the phylogenetic placement of these lineages suggests their pathogenic potential for humans and/or other mammals, this assessment is preliminary pending further clinical and ecological data. Clarifying the epizootiology of *Leptospira* in European bats would help to assess potential risks to public health and identify mitigation measures.

## Supplementary Material

Supplemental Material

## Data and materials availability

All data needed to evaluate the conclusions are present in the paper.
