# Supplementary material for: Detection of Leptospira species in bat cadavers, Czech and Slovak Republics
Source: Emerg Microbes Infect. 2022 Sep 26;11(1):2211–3. doi: 10.1080/22221751.2022.2117095 (PMC9518262; doi:10.1080/22221751.2022.2117095)
Supplement: Supplemental Material [file TEMI_A_2117095_SM8308.zip › Appendix 1.docx]

**APPENDIX 1**

**Table 1.** *Leptospira* detection from Central European bats. The number of carcasses positive for *Leptospira* DNA from each bat species over the total sampled are indicated separately for each country. For species with positive detections, the number of sequences from each *Leptospira* lineage are provided.

| **Bat species** | **Czech Republic** | **Slovak Republic** | **Total** |
| --- | --- | --- | --- |
| *Eptesicus nilssoni* | 0/1 | 0/0 | 0/1 |
| *Hypsugo savii* | 0/3 | 0/0 | 0/3 |
| *Myotis bechsteinii* | 0/2 | 0/0 | 0/2 |
| *Myotis daubentonii* | 0/3 | 0/0 | 0/3 |
| *Myotis emarginatus* | 0/1 | 0/0 | 0/1 |
| *Myotis myotis* | 11/185 (5.95%) | 0/2 | 11/187 (5.9%) |
| *Myotis naterreri* | 0/1 | 0/0 | 0/1 |
| *Nyctalus noctula* | 2/61 (3.3%) | 1/8 (12.5%) | 3/69 (4.3%) |
| *Pipistrellus pipistrellus* | 0/7 | 0/0 | 0/7 |
| *Plecotus auritus* | 0/6 | 0/0 | 0/6 |
| *Plecotus austriacus* | 0/3 | 0/0 | 0/3 |
| *Rhinolophus hipposideros* | 0/3 | 0/0 | 0/3 |
| *Vespertilio murinus* | 0/14 | 0/0 | 0/14 |
|  | 13/290 (4.5%) | 1/10 (10%) | 14/300 (4.7%) |
