# Supplementary material for: Detection of Leptospira species in bat cadavers, Czech and Slovak Republics
Source: Emerg Microbes Infect. 2022 Sep 26;11(1):2211–3. doi: 10.1080/22221751.2022.2117095 (PMC9518262; doi:10.1080/22221751.2022.2117095)
Supplement: Supplemental Material [file TEMI_A_2117095_SM8308.zip › Appendix 2.docx]

**APPENDIX 2**

To assess the phylogenetic relationships between *Leptospira* sequences amplified from bats in this study, we first compiled a database of *lipL32* and *flaB* sequences available on GenBank from canonical *Leptospira* species, from genomes of newly described species isolated from soil and water samples ﻿[1], and from previous studies of *Leptospira* in bats that used *lipL32* or *flaB* as the PCR target ﻿[2, 3, 4, 5, 6, 7]. Compiled sequences for each gene were aligned with MAFFT v7.490 using the local iterative L-INS-i method ﻿[8]. Simultaneous substitution model selection and maximum likelihood tree estimation was then implemented in IQ-TREE v2.1.1 ﻿[9]. The best substitution model for the aligned *lipL32* sequences according to the Bayesian information criterion (BIC) was a transition model with unequal base frequencies (estimated empirically), a proportion of invariable sites, and a discrete Gamma model with four rate categories to account for rate heterogeneity across sites (TIM3+F+I+G4). The best substitution model for the aligned *flaB* sequences according to BIC was a transversion model with equal base frequencies a proportion of invariable sites, and a discrete Gamma model with four rate categories to account for rate heterogeneity across sites (TVMe+I+G4). Branch support across trees for each gene was estimated with 1000 bootstrap iterations ﻿[10]. The phylogenetic trees were drawn in R v4.1.0 using the GGTREE ﻿[11, 12].

**References**

[1] Vincent AT, Schiettekatte O, Goarant C, et al. Revisiting the taxonomy and evolution of pathogenicity of the genus *Leptospira* through the prism of genomics. PLoS Negl Trop Dis. 2019;13(5):e0007270.

﻿[2] Ogawa H, Koizumi N, Ohnuma A, et al. Molecular epidemiology of pathogenic Leptospira spp. in the straw-colored fruit bat (*Eidolon helvum*) migrating to Zambia from the Democratic Republic of Congo. Infect Genet Evol2015;32:143–7.

﻿[3] Bai Y, Urushadze L, Osikowicz L, et al. Molecular survey of bacterial zoonotic agents in bats from the country of Georgia (Caucasus). PLoS One. 2017;12(1):e0171175.

﻿[4] Ballados-González GG, Sánchez-Montes S, Romero-Salas D, et al. Detection of pathogenic *Leptospira* species associated with phyllostomid bats (Mammalia: Chiroptera) from Veracruz, Mexico. Transbound Emerg Dis. 2018;65(3):773–81.

﻿﻿[5] Zhao M, Xiao X, Han H-J, et al. *Leptospira* in bats from Hubei Province, China, 2018. J Wildl Dis. 2019;55(4):940.

﻿[6] Mateus J, Gómez N, Herrera-Sepúlveda MT, Hidalgo M, et al. Bats are a potential reservoir of pathogenic *Leptospira* species in Colombia. J Infect Dev Ctries. 2019;13(4):278–83.

﻿[7] Seidlova V, Nemcova M, Pikula J, et al. Urinary shedding of leptospires in palearctic bats. Transbound Emerg Dis. 2021;68(6):3089–95.

﻿[8] Katoh K, Standley DM. MAFFT multiple sequence alignment software version 7: improvements in performance and usability. Mol Biol Evol. 2013 Apr 1;30(4):772–80.

﻿[9] Minh BQ, Schmidt HA, Chernomor O, et al. IQ-TREE 2: new models and efficient methods for phylogenetic inference in the genomic era. Mol Biol Evol. 2020;37(5):1530–4.

[10] Hoang DT, Chernomor O, von Haeseler A, et al. UFBoot2: improving the ultrafast bootstrap approximation. Mol Biol Evol. 2018;35(2):518–22.

﻿﻿ [11] Yu G, Smith DK, Zhu H, GGTREE: an R package for visualization and annotation of phylogenetic trees with their covariates and other associated data. Methods Ecol Evol. 2017;8(1):28–36.

[12] R Core Team. R: a language and environment for statistical computing. 2021. Available from: http://www.r-project.org
