# Supplementary material for: Detection of Leptospira species in bat cadavers, Czech and Slovak Republics
Source: Emerg Microbes Infect. 2022 Sep 26;11(1):2211–3. doi: 10.1080/22221751.2022.2117095 (PMC9518262; doi:10.1080/22221751.2022.2117095)
Supplement: Supplemental Material [file TEMI_A_2117095_SM8308.zip › Figures 1A and 1B captions.docx]

**Figure 1A.** Phylogenetic relationships between *Leptospira* *lipL32* sequences. Separate groups, including new sequences detected in Central European bats, are indicated by distinct symbols. The maximum likelihood tree was inferred using a TIM3+F+I+G4 model in IQ-TREE v2.1.1. Numbers next to nodes indicate the percent bootstrap support after 1000 replicates. Branch lengths are in units of substitutions per site.

**Figure 1B.** Phylogenetic relationships between *Leptospira* *flaB* sequences. Separate groups, including new sequences detected in Central European bats, are indicated by distinct symbols. The maximum likelihood tree was inferred using a TVMe+I+G4 model in IQ-TREE v2.1.1. Numbers next to nodes indicate the percent bootstrap support after 1000 replicates. Branch lengths are in units of substitutions per site.
